# Proposal of the Definition for COVID-19-Associated Coagulopathy

**DOI:** 10.3390/jcm10020191

**Published:** 2021-01-07

**Authors:** Toshiaki Iba, Theodore E. Warkentin, Jecko Thachil, Marcel Levi, Jerrold H. Levy

**Affiliations:** 1 Department of Emergency and Disaster Medicine, Juntendo University Graduate School of Medicine, Tokyo 113-8421, Japan; 2 Department of Pathology and Molecular Medicine, McMaster University, Hamilton, ON L8S 4L8, Canada; twarken@mcmaster.ca; 3 Department of Medicine, McMaster University, Hamilton, ON L8S 4L8, Canada; 4 Department of Haematology, Manchester Royal Infirmary, Manchester M13 9WL, UK; Jecko.Thachil@cmft.nhs.uk; 5 Department of Medicine, University College London Hospitals NHS Foundation Trust, and Cardio-Metabolic Programme-NIHR UCLH/UCL BRC, London NW1 2BU, UK; marcel.levi@nhs.net; 6 Department of Anesthesiology, Critical Care, and Surgery, Duke University School of Medicine, Durham, NC 27710, USA; jer-rold.levy@duke.edu

**Keywords:** COVID-19, disseminated intravascular coagulation, endothelial cell, coagulopathy, heparin

## Abstract

Thrombotic events are common complications in COVID-19 patients that include both thrombus formation in large vessels and the microvasculature of the lung and other organs. COVID-19-associated coagulopathy (CAC) and disseminated intravascular coagulation (DIC) have similarities and differences, and whether CAC is a form of DIC is the subject of debate. Reported mechanisms of CAC include activated coagulation, endotheliopathy, up-regulated innate and adaptive immunity, and activated complement system. Although the clinical features and laboratory findings of CAC and DIC seem different, there are fundamental similarities that should be considered. Basically, the pathological findings of COVID-19 fall within the scope of the definition of DIC, i.e., systemic activation of coagulation caused by or resulting from the microvascular damage. Therefore, we suggest that although CAC differs from usual infection-associated DIC, its various features indicate that it can be considered a thrombotic phenotype DIC. This review summarizes the current knowledge about CAC including differences and similarities with sepsis-associated DIC.

## 1. Introduction

**Figure 1:**
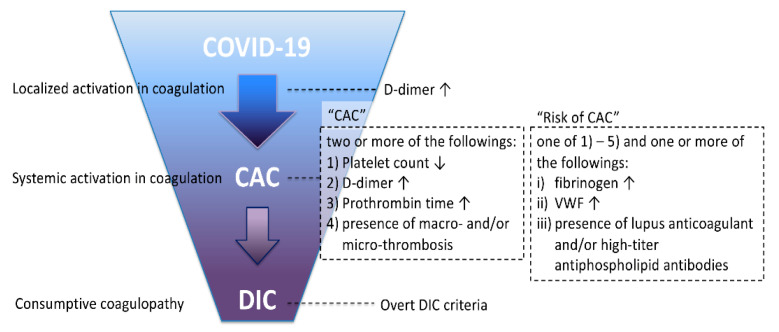
The progression from COVID-19-associated coagulopathy to disseminated intravascular coagulation (DIC). Severe Acute Respiratory Syndrome Coronavirus 2 (SARS-CoV-2) elicits thrombotic property by damaging the vascular endothelial cells. Activation in coagulation is initially localized in the lung microcirculation, however, when it expands systemically, it is called COVID-19-associated coagulopathy (CAC). The diagnostic criteria of CAC are proposed as (A) proven COVID-19 and (B) two or more of the following criteria: (1) decrease in platelet count (less than 150 × 10^9^/L); (2) increase in D-dimer (more than two times the upper limit of normal); (3) >1 s prolonged prothrombin time or International Normalized Ratio (INR) > 1.2; (4) decrease in fibrinogen level; (5) presence of thrombosis (macrothrombosis including deep vein thrombosis/venous thromboembolism, thrombotic stroke, acute coronary syndrome, limb artery thrombosis, mesenteric artery thrombosis, etc., and/or microthrombosis including skin, acral lesions, etc.). “Risk of CAC” is defined as one of above five criteria and one of following criteria: (i) increase in fibrinogen level; (ii) increased von Willebrand factor (VWF) (more than two times the upper normal limit); (iii) presence of lupus anticoagulant and/or high-titer antiphospholipid antibodies. CAC and risk of CAC can progress to disseminated intravascular coagulation (DIC) when the disease progresses.

COVID-19 is known to be frequently associated with coagulopathy and thrombotic complications. An initial report of COVID-19 patients with acute respiratory failure noted the development of disseminated intravascular coagulation (DIC) according to International Society on Thrombosis and Haemostasis (ISTH) criteria [1], with the incidence of DIC in non-survivors much higher than that in survivors (71.4% vs. 0.6%, respectively) [2]. In contrast, other studies reported the incidence of DIC to be low on initial patient presentation [3], findings that suggest COVID-19-associated coagulopathy (CAC) progresses to DIC as the disease progresses or due to secondary bacterial infections (Figure 1). Recent studies suggest the mechanisms of bacterial sepsis-induced coagulopathy (SIC) and that of CAC are different and more complicated in CAC [4]. Of note is that a decrease in platelet count and prolongation of prothrombin time—the hallmark of SIC—are uncommon in the early phase of COVID-19. However, the fundamental concept of DIC, as proposed by the ISTH in 2001 [1], is “an acquired syndrome characterized by the intravascular activation of coagulation with loss of localization arising from different causes. It can originate from and cause damage to the microvasculature, which, if sufficiently severe, can produce organ dysfunction”, and recent postmortem findings suggest that the histologic findings of COVID-19 are in accord with this definition [5]. Thus, we intend to compare the mechanisms of coagulopathy in COVID-19 to sepsis-associated DIC and its progenitor SIC from the point of this view.

## 2. Characteristic Differences between COVID-19-Associated Coagulopathy and Sepsis-Associated DIC

DIC is a laboratory and clinical diagnosis that arises from various underlying diseases. The clinical spectrum of DIC ranges from a thrombotic phenotype to a fibrinolytic phenotype, with intermediary mixed expressions [6]. A thrombotic phenotype of DIC and its major feature of multiorgan dysfunction is a common complication of bacterial sepsis [7]. Investigators have suggested that DIC is a consumptive coagulation disorder, and CAC is not DIC since bleeding is uncommon. However, bleeding events are also uncommon in many types of DIC that express a thrombotic phenotype and bleeding can occur in CAC with disease progression and includes intracranial hemorrhage and spontaneous hemorrhage. Al-Samkari et al. [8] reported overall and major bleeding rates were 4.8% (19/400) and 2.3% (3/144), respectively. Furthermore, postmortem examination noted both thrombi and hemorrhage [9]. As a matter of fact, the incidence of in situ pulmonary arterial thrombosis is higher in COVID-19 compared to that in sepsis-associated acute respiratory distress syndrome since SARS-CoV-2 infects the lung capillary endothelial cells causing cell death and loss of endothelial antithrombotic properties. Subsequently, microclots formed in the pulmonary capillaries extend into the proximal (arteriolar/arterial) side. Another important mechanism is the vasoconstriction by angiotensin II [10]. SARS-CoV-2 binds to angiotensin-converting enzyme 2 (ACE2) to enter the host cells and prevents its enzymatic activity to convert angiotensin II to angiotensin 1–7, contributing to vasoconstriction and clot formation [11].

## 3. Pathophysiology of COVID-19-Associated Coagulopathy and Bacterial Sepsis-Associated DIC

### 3.1. Endothelial Activation and Damage

**Figure 2:**
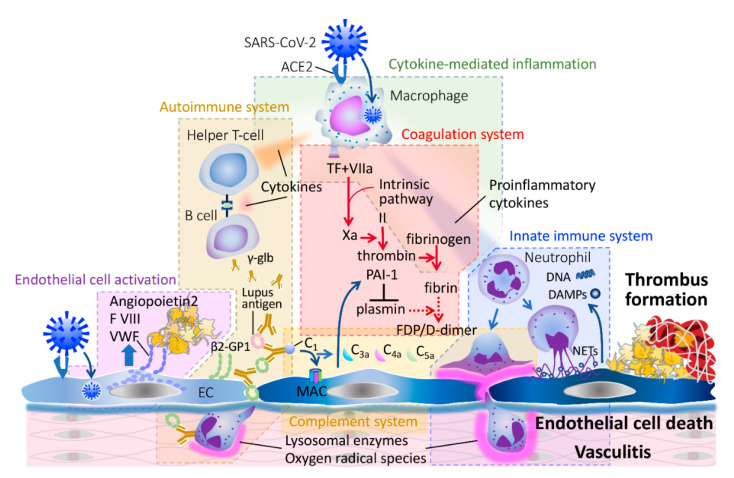
The mechanisms of COVID-19-associated coagulopathy. The pathogenesis of COVID-19-associated coagulopathy is complex. Activated coagulation and endothelial cell (EC) damage are the two main axes. SARS-CoV-2 infects macrophage and endothelial cells via binding to angiotensin 2 receptor (ACE2). Tissue factor (TF) expressed on immune cells and excess cytokine production stimulate coagulation and damages endothelial cells. Von Willebrand factor (VWF), factor VIII, and angiopoietin 2 are released from endothelial cells. Antiphospholipid antibodies such as lupus anticoagulant antibodies and anti-β_2_ glycoprotein (GP) I antibodies could initiate vasculitis. In addition, the complement system and innate immunity relate to the endothelial damage. The membrane attack complex (MAC) damages cellular membrane and activated neutrophil release neutrophil extracellular traps (NETs), damage-associated molecular patterns (DAMPs), and chemical mediators to damage the vasculature.

The direct SARS-CoV-2 infection of vascular endothelial cells is the unique characteristic of COVID-19, and this etiology may explain the extremely high incidence of thrombotic complications. As a result of endothelial infection, von Willebrand factor (VWF) stored in the Weibel–Palade body of the endothelial cells is released into the circulation, promoting platelet/vessel wall interactions, and subsequent platelet aggregation. The associated release of factor VIII can increase coagulability [10] (Figure 2). However, the increased VWF with a decrease of its cleaving protease ADAMTS13 (a disintegrin and metalloprotease with a thrombospondin type 1 motif, member 13) is not restricted to CAC but also occurs in sepsis-associated DIC [12]. It is known that the secretion of VWF is stimulated by various infectious stimuli, such as inflammatory cytokines, thrombin, and fibrin, leading to tethering of platelets to endothelial cells [13]. Although increased VWF initiated deposition of platelet-rich clots in the lung microcirculation is considered to be a major mechanism of respiratory failure in COVID-19 [14], this event is also seen in bacterial sepsis [15]. Of note is microcirculatory clot formation in the lung as an early event in COVID-19, but this also occurs as the result of systemic inflammation in septic patients with Acure Respiratory Distress Syndrome (ARDS) [16]. In CAC, clot formation is likely localized initially within the lungs, and only D-dimer levels elevate at this stage. However, in the advanced stage, the systemic activation in coagulation and disseminated microthrombosis occurs in the multiple organs.

Angiopoietin 2 is another important mediator also stored in Weibel–Palade bodies. The circulating levels of angiopoietin 2 are reported to increase in COVID-19 and induce procoagulant and proinflammatory reactions [17]. Angiopoietin 2 binds to its receptor Tie2 expressed on the endothelial cells competitively to angiopoietin 1 and abrogates anti-inflammatory, anticoagulatory, and antiapoptotic signaling via angiopoietin 1-Tie2 binding [18]. Again, angiopoietin 2 is known to stimulate prothrombotic responses through tissue factor and phosphatidylserine exposure in sepsis. Thus, angiopoietin 2 is considered as the common factor in the pathogenesis of CAC and sepsis-associated DIC.

### 3.2. Coagulation and Fibrinolytic Systems in Infection

Both inflammation and coagulation are critical host responses to infection and activation of coagulation in COVID-19 significantly overlaps with bacterial sepsis [4]. In SIC/sepsis-associated DIC, coagulation activation is initiated mainly through the tissue factor dependent pathway [7]. Tissue factor is expressed on macrophages/monocytes as well as activated neutrophils and damaged endothelial cells [19]. In addition, phosphatidylserine expressed on various damaged cells and microvesicles further activate the coagulation cascade [20]. The typical feature of SIC/sepsis-associated DIC is a thrombotic phenotype induced by excess plasminogen activator inhibitor-1 (PAI-1) release and subsequent fibrinolytic suppression. In contrast, the primary target of SARS-CoV-2 is the lung alveolar cells, and the local fibrinolysis in response to fibrin deposition is upregulated by intra-alveolar urokinase plasminogen activator (uPA)/uPA receptor system [21]. COVID-19 patients develop respiratory distress from endothelial injury, and the fibrin formation in the lung microcirculation manifests clinically as elevated D-dimer before progressing into a consumptive coagulopathy. The inflammation progresses systemically when COVID-19 patients deteriorate to require hospital admission, and the D-dimer elevations are manifested as a balance of activation in coagulation and suppression in fibrinolysis. After all, the variability of coagulation biomarkers, as reported in COVID-19 patients, represents the different time progressions of the disease state in patients as well as the difference between local and systemic inflammation.

### 3.3. Innate Immune System

The increase in inflammatory cytokine levels represents exacerbation of COVID-19 infection. Increased inflammatory cytokines can provoke a condition that mimics hemophagocytic lymphohistiocytosis (HLH)/macrophage activation syndrome (MAS), increasing procoagulant status [22]. Pathogenesis of HLH/MAS is estimated to be associated with increased activation of macrophages and natural killer cells. An autocrine loop in inflammatory cytokines (e.g., interleukin (IL)-1β, IL-6, IL-18, and interferon-γ) leads to cytokine-mediated dysregulated inflammation [23], and HLH/MAS occurs secondary to both viral and bacterial infections [23].

Neutrophils are also critical components of the innate immune system, with a role in activating coagulation in acute infection. The activated neutrophils induce coagulopathy/DIC via releasing damage-associated molecular patterns (DAMPs) and neutrophil extracellular traps (NETs) [19]. Activated neutrophils further adhere to and damage the endothelial cells and accelerate thrombus formation in sepsis. Middleton et al. [24] reported that NETs elicit thrombotic presentations in COVID-19 patients by triggering immunothrombosis.

### 3.4. Adaptive Immune System

Although the relevance to the coagulopathy has not been clarified, the presence of autoantibodies such as antiphospholipid antibodies is frequently seen in COVID-19 [25]. Antiphospholipid antibodies, including lupus anticoagulant, anticardiolipin, and anti-β_2_-glycoprotein (GP) I antibodies induce vasculitis following deposition of immune complexes in the vascular wall, leading to vascular complications. It is speculated the immune complex-induced damage (type 3 hypersensitivity) plays an important role in the development of autoimmune-related vascular injury that mimics Kawasaki disease and Guillain–Barré syndrome in COVID-19 [26]. Secondary antiphospholipid syndrome (APS) is rarely seen in bacterial sepsis, but it can be complicated by both arterial and venous thromboses. Wiedermann et al. [27] reported that the positive rate of antiphospholipid antibodies in septic patients with acute lung injury was low, although some show high titers. The role of antiphospholipid antibodies in CAC versus sepsis-associated DIC remains to be further determined.

### 3.5. Complement System

Coagulation and innate immunity are the essential mechanisms that orchestrate the host defense against invading pathogens, and the complement system plays an integral role between innate immunity and hemostatic pathway in sepsis. However, excess activation of the complement system is also known to be involved in the pathogenesis of host injury [28]. In COVID-19, the damage of endothelial cells occurs as a result of the autoimmune response and subsequently leads to tissue damage and thrombosis. Magro et al. [29] reported atypical hemolytic uremic syndrome-like condition, a syndrome with dysregulated activation in complement activation, may contribute to the pathogenesis of COVID-19-associated endotheliopathy. Presumably, the excess anaphylatoxins C3a, C4a, and C5a result in a proinflammatory and procoagulant environment. In addition, thrombogenic endotheliopathy with deposition of C5b-9 (membrane attack component, MAC) confirmed in the biopsy specimens obtained from COVID-19 patients has been reported [30]. The complement system has attracted attention as a therapeutic target, for example by administration of C1 inhibitor or by targeting (pre)kallikrein.

## 4. Diagnosis of CAC and DIC

The diagnosis of DIC in sepsis is usually made based on the diagnostic criteria established by ISTH. The ISTH overt-DIC diagnostic criteria consist of four laboratory findings, i.e., decreased platelet count, elevated fibrin-related biomarker (e.g., D-dimer), prolonged prothrombin time, and decreased fibrinogen level [1]. Among them, only D-dimer level is usually elevated in COVID-19 in its early stage [31]. In this phase, platelet count and prothrombin time are maintained in the normal range, and fibrinogen level is often elevated as an acute phase response to the inflammation, providing indirect evidence against a major component of consumptive coagulopathy. Hyperfibrinogenemia may contribute to hypercoagulability and assumed to be associated with increased mortality both in COVID-19 and sepsis [32,33].

The early phase of sepsis-induced coagulation disorder without fulminant consumptive coagulopathy is defined as SIC by ISTH, with the platelet count and prothrombin time comprising the two biomarkers used for defining SIC [34]. In CAC, elevated D-dimer level is reported to be an indicator of disease severity [2], but that response is variable in SIC [35]. In CAC, the fibrin formed in lung microcirculation is dissolved by upregulated fibrinolysis from uPA/uPA receptor system activation [21], while fibrinolysis is attenuated by increased production of PAI-1 in SIC/DIC and D-dimer elevation accordingly is suppressed [36]. As a result, CAC demonstrates different laboratory findings from SIC and overt DIC. However, it should be realized that one criterion does not fit to all coagulopathies arising from different triggers. Together with the progress in further defining various types of DIC, specific criteria continue to be developed [37]. Levi et al. [38] described “one of the pathogenic hallmarks of DIC is dysregulated thrombin generation…there is not yet definitive proof of excessive thrombin generation in these COVID-19 patients.” Recently, Goshua et al. [10] showed a more intense increase in thrombin-antithrombin complex (TAT) in COVID-19 ICU patients compared to non-ICU patients and this finding will support the idea that thrombin generation plays a role in the pathogenesis of CAC.

The official definition of CAC has not been established yet but should be considered for both research and therapeutic considerations. For this reason, we propose CAC may be defined as a proven COVID-19 test with two or more of the following four criteria: (1) decrease in platelet count (less than 150 × 10^9^/L); (2) increase in D-dimer (more than two times the upper limit of normal); (3) >1 s prolonged prothrombin time or INR > 1.2; (4) presence of thrombosis (macrothrombosis including deep vein thrombosis/venous thromboembolism, thrombotic stroke, acute coronary syndrome, limb artery thrombosis, mesenteric artery thrombosis, etc., and/or microthrombosis including skin, acral lesions, etc.); and if the patient meets one of the above 4 criteria and also one or more of following criteria: (i) increase in fibrinogen level; (ii) increased VWF (more than two times the upper normal limit); (iii) presence of lupus anticoagulant and/or high-titer antiphospholipid antibodies, they are defined as “risk of CAC”. By this definition, CAC was recognized in less than 10% of the patients on admission, however, the incidence was more than 60% when the patients transferred to the ICU without meeting criteria for overt DIC.

## 5. Skin and Acral Lesions

COVID-19 shows a variety of skin and acral manifestations from erythema to ischemic limb necrosis. The most common cutaneous manifestation is maculopapular exanthem, followed by a papulovesicular rash, urticaria, painful acral red-purple papules, livedo reticularis, and petechiae. The majority of lesions were localized on the trunk, but approximately 20% of patients experienced cutaneous manifestations in the hands and feet that are typically known as COVID toes [39]. The typical COVID toes show the erythematous to purpuric macules, papules, and/or vesicles. These skin manifestations often coexist with acro-ischemic changes, and the acral lesions often occurred as a late-phase manifestation of COVID-19 [39]. It is still unclear whether these changes are the result of the viral cytopathic process or the host reactions to the infection, but histological examination demonstrated focal fibrinoid necrosis and thrombotic occlusive vasculopathy of the artery [40]. Skin necrosis and dermal manifestation, accompanied by arterial occlusion is relatively rare in septic patients, but limb and digit ischemic necrosis can occur and is termed acrocyanosis or symmetrical peripheral gangrene. Of note, ischemic limb injury in septic shock is explained by concurrent DIC and depletion of natural anticoagulant factors (protein C, antithrombin) often as a result of concomitant acute ischemic hepatitis (shock liver) [41]. Whether the skin and acral lesions in COVID-19 and sepsis-associated DIC have overlapping pathophysiologic features is still unknown, and further study is warranted.

## 6. Anticoagulant Therapy Using Heparin for CAC and DIC

Anticoagulant therapy using low-molecular-weight heparin (LMWH) is widely accepted as the standard therapy for COVID-19 patients [42]. Although the result of the prospective randomized study has not been achieved yet, the effect of heparins, mainly LMWH, is reported in the retrospective studies. In contrast, the effect of heparin on sepsis is still controversial. Murao et al. [43] performed a meta-analysis with regard to the effect of unfractionated heparin (UFH) and LMWH for sepsis and reported that though three studies showed potential survival benefits in patients with sepsis, trials with low risk of bias were lacking, and the overall impact remains unclear. From these results, it is assumed that anticoagulation using heparins are possibly beneficial both in sepsis and COVID-19. However, further investigation is demanded to confirm the beneficial effect.

## 7. Conclusions

Sepsis following an acute infection can be complicated by DIC. There are both similarities and differences between sepsis-associated DIC and CAC, with the latter disorder often not meeting DIC criteria despite numerous coagulation laboratory abnormalities and high frequency of microvascular and macrovascular thrombosis. The changes of laboratory biomarkers differ since fibrin formation in CAC is initially localized; however, laboratory features overlap with DIC as the disease progresses. The pathophysiology of DIC and CAC include endothelial activation and damage, changes in coagulation and fibrinolysis, and with similar host responses to the infection. Furthermore, the pathological findings of COVID-19 match the definition of DIC. Therefore, we suggest that CAC shows the pattern of a thrombotic phenotype DIC, and propose its own distinct criteria to better define the concept of “CAC”.
